# Monthly Summary

**Published:** 1890-11

**Authors:** 


### Monthly Summary.

PRIMARY ANÆSTHESIA IN MINOR OPERATIONS.—Dr. F. B. Harrington, M. D., in the *Boston Medical and Surgical Journal*, refers to the need of an anæsthetic which shall be suitable for minor surgical operations of short duration, and in which the after effects shall be but trifling. He believes that the brief duration of nitrous oxide anæsthesia makes it unsuitable for minor surgical operations, although it is rapid and safe of application. Chloroform he has never used. Cocaine, though of use in many cases, is not without danger and sometimes fails. Profound ether anæsthesia requires time and previous preparation of the patient, and the after-effects are often very tedious. He has for these reasons made use of

"primary ether anæsthesia for the last three years in several hundred surgical cases, and has never seen any ill effects from the use of ether in this way. The method has been perfectly satisfactory in over 90 per cent. of the cases. Vomiting rarely occurs. Statistics of his cases show that from 6 to 73 inhalations were necessary for the production of anæsthesia, occupying from 20 seconds to 7 minutes; the period of insensibility lasted from 25 seconds to 6 minutes. The following is his description of the method of procedure he has adopted:

As far as is possible in a short time, the confidence of the patient is gained. They are told that a few breaths will make them insensible to pain and that in a few minutes they will be in a condition to go home. Women and children are, as a rule, less alarmed if they are allowed to inhale the ether in a sitting posture. The effort of keeping erect takes the attention and the position is less suggestive of complete helplessness. This is not a desirable position for strong men unless they are held in by a strap. It is well always to have assistants at hand, although a large number of cases might easily be handled alone. An ounce or more of ether is poured on a sponge or towel. This is given to the patient with directions to breathe rapidly and deeply.

It is important to teach the patient how he is to breathe before applying the ether. I tell them to breathe as a dog pants. The rapid and moderately deep breath I think is the best one, not only for rapid primary anæsthesia, but also for the induction of profound anæsthesia. It is a well-known fact that rapid breathing of air itself produces a certain degree of anæsthesia. Another reason for rapid breathing is that there is a tendency to continue any muscular act in an automatic way after consciousness of the act has ceased. We see this in the repeated use of certain words or phrases, or the continuance of a motion of the leg or arm. I often see patients wholly unconscious of pain breathing at the rate of 40 per minute, the impulse to such breathing having been given during consciousness.

The tendency is naturally toward slow breathing and much more time is required if the breathing be slow. The sponge should be held a short distance from the face and should not cause coughing.

After a dozen or more rapid breaths the operator asks the patient if he is ready. This is repeated until the patient answers "yes" or there is no response. If but an incision or two is necessary, the ether is at once removed; if more time is required the ether is applied for a few seconds longer.—*Med. Review.*

---

EUPHORINE.—Under the name of euphorine Dr. Sansoni describes the physical and therapeutical properties of a new compound which, chemically, is phenylurethan. It is a white crystalline powder, with a faint aromatic odor and a slight taste suggesting that of cloves. It is sparingly soluble in cold water, readily dissolves in alcohol, and is best administered in white wine, in which it is easily dissolved. Given to healthy men, in doses of from 1½ to 3 grains, euphorine has no apparent effect upon the pulse, the respiration, or the temperature; and in dogs large doses do not lower the blood-pressure, as determined by the manometer. Still more important is the fact that it causes no changes in the blood—in animals the blood remaining normal even after toxic doses have been given. Clinically, Sansoni finds that euphorine possesses valuable antipyretic, antiseptic, antirheumatic, and, in some cases, analgesic effects. As an antipyretic, he has employed the drug in fever from various causes, such as typhoid fever, croupous pneumonia, phthisis, acute rheumatism, pleurisy, orchitis, influenza, etc., administering it either as a powder or dissolved in wine. The fall of temperature begins within an hour after the administration, reaches its maximum usually in about three hours, and continues, as a rule, for from five to seven hours. In some cases the fall is more brief, and in a few it continues for fourteen hours. The subsequent rise of temperature is usually sudden and accompanied with a chill, the duration and severity of which are proportionate to the intensity of the disease. During the period of apyrexia the patient has a feeling of well-being. Cyanosis is seldom observed, and the pulse and respiration are regular. The antipyretic effect varies in different persons, and it is best to begin with a small dose (1½ grain), increasing until the proper amount is determined. To most adults, from 15 to 22 grains can be given daily without producing harmful effects, its antithermic power being about twice that of antipyrine. In rheumatism, both acute and chronic, the doses of euphorine should be larger than those given to reduce temperature. From 22 to 30 grains, or even more, should be given daily. In acute rheumatism the good effects appear very soon after the drug has been administered; the temperature falls, and the pain, whether due to inflammation or to the pressure of the swelling, disappears. As an analgetic, euphorine is very useful in orchitis, less so in sciatica, lightning pains of tabes, and trigeminal neuralgia, and is almost useless in intercostal neuralgia and migraine. The observations that carbolic acid is formed, when euphorine is brought in contact with an alkali, led Sansoni to test its antiseptic powers, which, in the case of an obstinate ulcer and one of chronic ophthalmia, he found were excellent.—*Medical News*, October 11, 1890.

---

WANDERING RASH ON CHILDREN'S TONGUES.—At a recent meeting of the Calcutta Medical Society, Dr. Towers showed a case of this kind. He said it was a condition of the dorsum of the tongue which has only of late years been differentiated as a distinct affection. Other names for it are "ringworm, circular exfoliations, geographical tongue, etc." It is well described and depicted in Butin's "Diseases of the Tongue." The characters of the rash are, that it is met with chiefly in children, that it seldom gives rise to disagreeable symptoms, that it is unaffected by treatment, that its area and distribution are subject to constant changes, that it is apt to persist for a long time, and ultimately to disappear spontaneously. It consists of one or more circinate patches on the dorsum, of various sizes, separate, or confident, smooth, red, and on the same level as the surrounding surface, with a distinct whitish or yellowish border. The filiform papillæ have been shed, but the fungiform distinctly remain. Rings may form within rings. It commences always on the dorsum. On account of the few subjective symptoms it is usually discovered by accident. The cause of the disease is unknown, but it appears to be associated with debility. It has to be differentiated from syphilitic mucous patches, leucoma, and icthyosis. In syphilis the patches are distinctly raised above the surrounding surface and are grayish upon their surface, and there are present other signs of syphilis. Leucoma is a disease of adults, and consists of pearly patches with red borders and bases. Moreover, leucomatous patches do not change from day to day. Icthyosis is also a disease of adults, and consists of patches of hypertrophied filiform papillæ, while just the opposite condition obtains in this affection.—*Indian Medical Gazette.*

---

A LOCAL ANÆSTHETIC FORMULA.—An Exchange says: Local anæsthesia is produced at one of the leading hospitals by means of a spray composed of 10 parts of chloroform, 15 parts of ether and 1 part of methol. After one minute's application of this compound spray, complete anæsthesia of the skin and neighboring tissues is produced and will persist from 2 to 6 minutes. This suffices for some minor operations, such as openining an abscess of the cervical glands, incising a deep-seated whitlow, or excising an epithelioma of the nose, etc.

---

NORMAL WEIGHT OF MAN.—A rule to determine the normal weight of man is as follows: A man should weigh just as many kilogrammes as he measures centimeters in height, after deducting one meter. A man who measures in height 1 meter 80 centimeters (5 ft. 11 in.) should weigh 80 kilogrammes, or about 160 pounds. The rule is both ingenious and approximately correct.

DANGERS OF SALIVATION FROM CALOMEL AND ACIDS.— 1. All the alkaline chlorides are incompatible with calomel, and we should, therefore, forbid all salt food after a dose of calomel has been given.

2. In certain diseases calomel is prescribed in fractional doses at the same time that the patient is taking iodide of potassium; now, as this salt causes the conversion of chloride into the bichloride, it is the wiser course not to have the two drugs together in the stomach.

3. Calomel should never be prescribed in the same mixture with preparations containing hydrocyanic acid, such as cherry-laurel water and the like.

Some forbid all acid drinks after a dose of calomel has been taken; but as acids convert this salt into the bichloride only when they are in concentrated hot solutions, this precaution seems hardly worth observing.—*Med. Record.*

---

COCAINE ANÆSTHESIA OBTAINED BY MEANS OF CATAPHORESIS.—Dr. Arthur Harreis (*Lancet*, October 25, 1890) writes that where local anæsthesia is required, cocaine hydrochlorate, administered by means of cataphoresis instead of hypodermically, should be employed. In a number of cases in which he adopted this method the anæsthesia was complete, and toxic symptoms of the drug were not observed. He uses a 10 per cent. solution of cocaine, with which the flannel-padded positive electrode, corresponding to the size of the area to be anæsthetized, is saturated. The large negative electrode is soaked in salt solution and placed in a suitable position on the surface of the body. A continuous current of twenty-five milliamperes is then passed for forty minutes. Dr. Harries believes that the causes of failure with this method are: That the currents used are too weak, and applied for too brief a time; that the operator does not understand the apparatus, and that a reserved current is used.—*Med. News.*

